# Small Extracellular Vesicle-Derived Circular RNA hsa_circ_0007386 as a Biomarker for the Diagnosis of Pleural Mesothelioma

**DOI:** 10.3390/cells13121037

**Published:** 2024-06-14

**Authors:** Sareh Zhand, Jiayan Liao, Alessandro Castorina, Man-Lee Yuen, Majid Ebrahimi Warkiani, Yuen-Yee Cheng

**Affiliations:** 1 School of Biomedical Engineering, University of Technology Sydney, Sydney, NSW 2007, Australia; 2 Institute for Biomedical Materials and Devices, Faculty of Science, University of Technology Sydney, Sydney, NSW 2007, Australia; 3 Laboratory of Cellular and Molecular Neuroscience (LCMN), School of Life Sciences, Faculty of Science, University of Technology Sydney, Sydney, NSW 2007, Australia; 4 Institute of Molecular Medicine, Sechenov First Moscow State University, Moscow 119991, Russia

**Keywords:** pleural mesothelioma, lung disease, small extracellular vesicle, circular RNA, digital PCR, diagnosis of pleural mesothelioma

## Abstract

Pleural mesothelioma (PM) is a highly aggressive tumor that is caused by asbestos exposure and lacks effective therapeutic regimens. Current procedures for PM diagnosis are invasive and can take a long time to reach a definitive result. Small extracellular vesicles (sEVs) have been identified as important communicators between tumor cells and their microenvironment via their cargo including circular RNAs (circRNAs). CircRNAs are thermodynamically stable, highly conserved, and have been found to be dysregulated in cancer. This study aimed to identify potential biomarkers for PM diagnosis by investigating the expression of specific circRNA gene pattern (hsa_circ_0007386) in cells and sEVs using digital polymerase chain reaction (dPCR). For this reason, 5 PM, 14 non-PM, and one normal mesothelial cell line were cultured. The sEV was isolated from the cells using the gold standard ultracentrifuge method. The RNA was extracted from both cells and sEVs, cDNA was synthesized, and dPCR was run. Results showed that hsa_circ_0007386 was significantly overexpressed in PM cell lines and sEVs compared to non-PM and normal mesothelial cell lines (*p* < 0.0001). The upregulation of hsa_circ_0007386 in PM highlights its potential as a diagnostic biomarker. This study underscores the importance and potential of circRNAs and sEVs as cancer diagnostic tools.

## 1. Introduction

Pleural mesothelioma (PM) is an aggressive cancer of the lung lining that occurs as a result of previous asbestos exposure either occupationally or environmentally [1]. The lack of specific biomarkers and different pathologic subtypes increase the difficulty of diagnosis and treatment. PM patients are usually diagnosed at an advanced stage and are usually short-lived with median survival ranging from 4 to 18 months [2,3] and five year survival rate currently stands at less than 5% [4]. Additionally, therapeutic options for PM patients who are not eligible for surgery are very limited [5]. There are three main histological subtypes of PM: epithelioid, biphasic, and sarcomatoid [6]. While epithelioid PM shows a morphology similar to normal pleura, biphasic and sarcomatoid subtypes are associated with a worse prognosis than the epithelioid subtype [7]. The PM exhibits a highly secretory cell type, releasing factors that can exert autocrine or paracrine effects on nearby tumor and stromal cells. These released factors likely contribute to the modulation of the extracellular environment and could potentially serve as a source for identifying cancer biomarkers [8]. Current procedures for PM diagnosis are invasive and can take a long time to reach a definitive result [9,10,11]. As the incidence of PM continues to rise, there is a pressing need for novel biomarkers for early detection. These tools and biomarkers are crucial for effective clinical management of PM, facilitating early diagnosis, monitoring prognosis, and predicting treatment outcomes [12].

In recent times, extracellular vesicles (EVs) have garnered attention as significant messengers facilitating communication between tumor cells and their surrounding microenvironment. These extracellular vesicles have emerged as key mediators in cancer biology, orchestrating cellular communication crucial to tumorigenesis and harboring distinctive cancer biomarker profiles [13]. EVs stand as promising candidates for both diagnosis and therapy owing to their detectability in non-invasive blood samples and other body fluids. This characteristic suggests significant potential for their application in diagnostic and therapeutic endeavors. The EVs released from mesothelioma cells provide crucial insights into the molecules and signaling pathways pivotal in the initiation and advancement of the tumor [14]. As delineated by the International Society of Extracellular Vesicles (ISEV), the designation “small extracellular vesicles” is the recommended nomenclature for the diverse array of vesicles derived from cell culture supernatants or physiological fluids [15,16]. EVs, particularly small extracellular vesicles (sEVs), represent a heterogeneous population of lipid-bilayer-delimited particles released from cells that are classified based on size [17,18,19]. These nano-sized particles (30–150 nm) originating from the endosomal pathway, play essential roles in both physiological and pathological phenomena, (22, 23), encompassing crucial functions in immune response [20], signal transduction [21], as well as tumor initiation, progression, invasion, and metastasis [22,23]. These vesicles exhibit enrichment in a repertoire of shared structural and functional proteins, including tetraspanins (CD9, CD81, CD82, and CD63) [24], Rab GTPase, and HSP90 (heat shock protein 90), among others [25]. Furthermore, sEVs transport a diverse cargo comprising proteins, nucleic acids (mRNA, regulatory RNA, and DNA components), lipids, metabolites, and organelles from donor cells. Recent findings suggest that these reservoirs of biomarkers hold promise for advancing early cancer detection, prognostication, and monitoring tumor progression [26,27,28]. In recent investigations, it has been established that Osteopontin, Galectin-1, Mesothelin, and VEGF exhibit elevated concentrations in sEVs derived from PM patients’ effusions in comparison to those from the benign group [14]. Furthermore, another study revealed increased expression levels of Galectin-1, Mesothelin, Osteopontin, and VEGF in PM patients relative to benign patients [15]. Some EV biomarkers were found to be abundant in the serum of mice exposed to asbestos, as well as elevated levels of exosomal ceruloplasmin, haptoglobin, and fibulin-1 [16]. The sEVs represent a largely untapped resource of diagnostic, prognostic, and predictive biomarkers with significant potential for clinical applications. [18,29,30].

**Figure 1:**
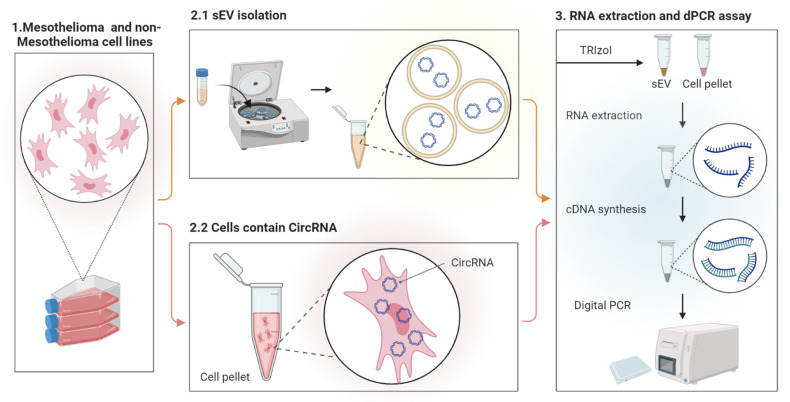
Schematic diagram showing an overview of the circRNA detection procedure using cultured cells as the starting material. In step 1, the PM, non-PM, and normal mesothelium cell lines were cultured in a specific culture medium. In step 2.1, sEVs were isolated from a cell culture-conditioned medium using ultracentrifuge and characterized. In step 2.2, the cells containing the desired CircRNA (hsa_circ_0007386) were harvested and the cell pellet isolated. In step 3, total RNA was extracted from the isolated sEVs and the cell pellets, cDNA was reverse transcribed from the total RNA using random hexamer, and finally, the copy number of hsa_circ_0007386 circRNA in the samples was measured using the digital PCR assay using specific designed primer/probe sets.

Circular RNA (circRNA) presents a distinct form of non-coding RNA characterized by its closed single-stranded structure lacking 5′ caps or 3′ poly(A) tails, setting it apart from linear messenger RNA (mRNA) [31]. They are originating from pre-mRNAs through a process termed back splicing [32]. CircRNAs exhibit remarkable stability in the bloodstream and possess a longer half-life compared to linear RNAs. Additionally, they exhibit resistance to exonuclease-mediated degradation, rendering them highly attractive candidates for blood-based diagnostic applications [33]. Recent investigations have unveiled a correlation between circRNA overexpression and tumorigenesis across various cancers, encompassing lung malignancies (including PM), liver, breast, prostate, bladder, colorectal, ovarian, central nervous system, stomach, as well as diverse hematological malignancies [34]. Published findings indicate a significant enrichment of circRNAs within sEVs, with levels exceedingly at least two-fold those observed in parental cells [35]. Recent studies highlighted the abundance and stability of circular RNAs within sEVs, suggesting their persistent functionality after uptaking by neighboring cells [35]. Exo-circRNAs produced by tumors are released into bodily fluids, where they exert effects on various aspects including diagnosis, suppression of metastasis, and induction of tumor cell apoptosis [36]. It has been suggested that cells may transfer circRNAs by excreting them in sEVs, acting as messengers in cell-to-cell communication, and studies also propose that the clearance of intracellular circRNAs may be associated with sEVs [37]. There is currently limited knowledge on circRNA expression in PM [38] and CircRNA expression in sEV derived from PM has not been explored yet. This study aims to use the digital PCR (dPCR) for accurate detection of the most frequently upregulated circRNA (hsa_circ_0007386) in sEVs derived from PM, non-PM, and normal mesothelial cell lines and compare the expression of this specific circRNA with non-PM and normal mesothelial cell lines to determine its suitability as a potential biomarker candidate for the early diagnosis of PM (Figure 1).

## 2. Materials and Methods

### 2.1. Reagents

The list of following antibodies including purified anti-human CD63 (Clone H5C6), purified anti-human CD9 (Clone HI9a), and purified anti-human CD81 (Clone 5A6) antibodies, along with the secondary antibody HRP-goat anti-mouse IgG (405306), were purchased from BioLegend (San Diego, CA, USA). BenchMark™ Pre-stained Protein Ladder (10748010) and Novex™ Sharp Pre-Stained Protein Standard (LC5800) were obtained from ThermoFisher (Waltham, MA, USA). RIPA Lysis and Extraction Buffer (89900), Pierce BCA protein assay kit (23227), 4X Bolt™ LDS Sample Buffer (B0007), polyvinylidene difluoride (PVDF) transfer membranes (88585), Bolt™ 4-12% Bis-Tris Plus Gels (NW04120BOX), Glycogen (R0561), and SuperSignal™ West Dura Extended Duration Substrate (37071) were purchased from Life Technologies (Waltham, MA, USA). TRIzol LS (10296010, Invitrogen, Carlsbad, CA, USA), phosphate buffered saline (PBS), Bovine serum albumins (A3059-10G), Chloroform (288306-1L), Isopropanol (I9516-500ML), and Ethyl alcohol (E7023) were purchased from Sigma Aldrich (Burlington, MA, USA). High-Capacity cDNA Reverse Transcription Kit (4368813) was purchased from Applied Biosystems (Foster City, CA, USA). The QIAcuity Probe PCR Kit (250102) and QIAcuity Nanoplate 26k 24-well (250001) were purchased from Qiagen (Hilden, Germany).

### 2.2. Cell Culture

A total of five human-established mesothelioma cell lines including (NCI-H28, NCI-H226, H2052, MSTO-211H, H2452), sourced from American Type Culture Collection (ATCC, Manassas, VA, USA), and 15 non-mesothelioma cancer cell lines including human lung cancer (H460, H1975, H3122), human gastric cancer (MKN45, KATO III), human colorectal cancer (HCT115, HCT116), human breast cancer (MCF-7), human melanoma (A375), human liver cancer (HepG2), human prostate cancer (LnCap), the normal mesothelium cell line (MeT5A), the microglial cell line (BV-2) sourced from American Type Culture Collection (ATCC), and the human melanoma (Colo679, Colo794) cell lines were sourced from Cell Bank Australia were used in this study. The cell lines repeatedly tested negative for mycoplasma in-house at UTS. It is worth mentioning that the PM cell lines used in the study are commercially available as an in vitro model of PM. All cells were maintained in RPMI 1640 Gibco (ThermoFisher, Waltham, MA, USA) supplemented with 10% (*v*/*v*) fetal calf serum (FCS) Gibco (ThermoFisher, Waltham, MA, USA), 100 U/mL penicillin and 100 mg/mL streptomycin Gibco (ThermoFisher, Waltham, MA, USA) in a T25 tissue culture flask (ThermoFisher, Waltham, MA, USA). The cultures were maintained at 37 °C in a humidified incubator with 5% CO_2_ and for the BV-2 cell line the RPMI 1460 was replaced with DMEM: F12.

### 2.3. Total RNA Isolation

The comprehensive procedure for RNA isolation has been previously documented in our publication [39]. In brief, approximately 1 × 10^6^ cell pellets were utilized for the isolation of total RNA using the TRIzol extraction method. Following the manufacturer’s protocol, 750 µL of TRIzol Reagent was combined with 250 µL of cell pellets, and the mixture was homogenized through repeated pipetting. After a 5 min incubation to ensure complete dissociation of the nucleoprotein complex, 200 µL of chloroform was added for lysis and incubated at room temperature for 2–3 min. Subsequently, the samples underwent centrifugation at 12,000× *g* for 15 min at 4 °C, resulting in phase separation. The upper aqueous phase, containing the RNA, was transferred to a new tube, and 500 µL of isopropanol along with 15 µg of Glycogen was added, followed by a 10 min incubation. After centrifugation at 12,000× *g* for 10 min at 4 °C, the RNA precipitate formed a white gel-like pellet at the bottom of the tube, and the supernatant was discarded. The pellet was then resuspended in 1 mL of 75% ethanol and centrifuged at 7500× *g* for 5 min at 4 °C. Following removal of the supernatant, the RNA pellet was air dried for 5–10 min, then resuspended in 20 µL of RNase-free water and incubated in a heat block at 60 °C for 10 min. Finally, the RNA samples were stored at −70 °C for subsequent use. Quantification of the extracted RNA samples was performed using the NanoDrop™ One Microvolume UV-Vis Spectrophotometer (Thermo Scientific, Waltham, MA, USA).

### 2.4. cDNA Synthesis

Then, 1 µg of total RNA was subjected to first-strand complementary DNA (cDNA) synthesis using a high-capacity cDNA reverse transcription kit (4368813, Applied Biosystems (Foster City, CA, USA) consisting of random hexamers and additional 50 µM of Oligo dT primer, in the following conditions: primer annealing at 25 °C for 10 min, cDNA synthesis at 37 °C for 2 h, denaturing at 85 °C for 5 min and for 1 cycle using a CFX96 PCR system (Bio-Rad, Hercules, CA, USA). The cDNA was stored at −20 °C for further experiments.

### 2.5. CircRNA Detection via Digital PCR

The digital PCR was performed using the QIAcuity Probe PCR kit (250102, Qiagen, Hilden, Germany) according to the manufacturer’s protocol. 1X concentration of probe PCR master mix, 0.8 μM forward primer (hsa_circ_0007386), 0.8 μM reverse primer (hsa_circ_0007386), and 0.4 μM probe (hsa_circ_0007386), 5 µL of each synthesized cDNA sample in a total volume of 40 μL was added to the Nanoplate 26k Qiagen (Hilden, Germany). Similarly, Amplification conditions consisted of 1 cycle of 95 °C for 2 min following 40 cycles of 95 °C for 15 s, and 60 °C for 30 s with plate read. The cycling and detection were performed on the QIAcuity digital PCR system. All experiments were normalized with respective hsa_circ_0000284 as a reference gene expression. Hsa_circ_0000284 was utilized as a reference gene for this study in order to normalize the detected circRNA copy number variation for each tested sample. The prime/probes used in the study are listed in our previous work [38] as well as in Table 1.

### 2.6. Preparation of Conditioned Medium (CM)

The comprehensive procedure for preparing CM has been previously documented in our earlier publication [40]. Briefly, for CM preparation from the cell lines, the cells were initially cultured in T175 tissue culture flasks (ThermoFisher, Waltham, MA, USA). Upon reaching approximately 70% confluency, equivalent to approximately 3 × 10^8^ cells, the supernatant was carefully removed, and the cells underwent two washes with phosphate-buffered saline (PBS). Subsequently, the cells were transferred to a sEV-free medium (RPMI 1640/DMEM: F12 without FCS) and cultured for 48 h at 37 °C in a humidified incubator set at 5% CO_2_ and 2% O_2_ (hypoxic conditions). Following incubation, the culture medium was collected for further experimentation.

### 2.7. Small EV Isolation from CM Using Ultracentrifugation

The comprehensive procedure for sEV isolation has been previously documented in our publication [39]. The media containing released EVs was initially subjected to centrifugation steps to eliminate cellular debris. This process involved centrifugation at 300× *g* (19,776 rotor, Sigma, Burlington, MA, USA) for 10 min to remove dead cells and debris, followed by a subsequent centrifugation at 2000× *g* (19,776 rotor, Sigma, Burlington, MA, USA) for 20 min to further eliminate debris. The resulting cell-free supernatant was then transferred to a new tube and centrifuged at 10,000× *g* (19,776 rotor, Sigma, Burlington, MA, USA) for 30 min to pellet microvesicles. The supernatant containing EVs was subsequently filtered through a sterile 0.22 µm syringe filter (Merck Millipore, Burlington, MA, USA) and subjected to ultracentrifugation at 100,000× *g* for 120 min (F37L Ti rotor, Beckman Coulter, Brea, CA, USA) to pellet small EVs (sEVs). After the removal of the supernatant, pellets containing EVs and contaminating proteins were re-suspended in a separate ultracentrifuge tube in phosphate-buffered saline (PBS) and centrifuged again at 100,000× *g* for 120 min. The supernatant was discarded, and the pellet was resuspended in 500 µL of PBS, previously filtered through a 0.22 µm syringe filter (WHA9913-2502, Sigma Aldrich, Burlington, MA, USA). The isolated sEVs were stored at −80 °C until further use. All centrifugation steps were conducted at 4 °C.

### 2.8. Nanoparticle Tracking Analysis

For nanoparticle tracking analysis (NTA), a ZetaView^®^ PMX-420 QUATT system (Particle Metrix, Munich, Germany) equipped with a 532 nm green laser was employed to determine the concentration and size distribution of sEVs. Isolated sEV samples (10 µL) were diluted to 990 µL using freshly filtered PBS (0.22 µm filter) (Dilution factor 1:100) and injected into the detection chamber via syringe. The camera settings, including a slider shutter at 650 and slider gain at 50, were manually adjusted and maintained consistently across all samples. Videos lasting 30 s were recorded, with 5 captures per sample. The detection threshold was set at 6, while blur and max jump distance were automatically configured. The temperature was maintained at 25 °C throughout the analysis. Data analysis was conducted using the NTA software (version 8.05.14 SP7).

### 2.9. Western Blot

The comprehensive procedure for Western Blotting for sEV has been previously documented in our publication [40]. In brief, to assess the purity of sEV isolation, sEVs derived from mesothelioma and non-mesothelioma cancer cells were lysed by adding an equal volume of RIPA lysis and extraction buffer (ThermoFisher, Waltham, MA, USA). The protein concentration of the sEVs was quantified using a Pierce BCA protein assay kit (Pierce Biotechnology, Rockford, IL, USA), following the manufacturer’s protocol. For Western blot analysis, sEV proteins (2 × 10^8^ particles; ~5 µg) were separated using Bolt™ 4–12% Bis-Tris Plus Gels (Invitrogen, USA). Samples were diluted in 4× Bolt™ LDS Sample Buffer (ThermoFisher, Waltham, MA, USA) and heated at 70 °C for 10 min before transfer onto polyvinylidene difluoride (PVDF) membranes (ThermoFisher, Waltham, MA, USA). The PVDF membrane was blocked with 5% non-fat powdered milk in PBS-T (PBS and 0.5% Tween-20) for one hour at room temperature, followed by overnight incubation at 4 °C with primary antibodies against human CD63, CD9, and CD81 (1:500 in PBS-T) separately. Subsequently, the blots were incubated with an appropriate HRP-conjugated goat anti-mouse IgG secondary antibody (1:2000) in PBS-T for 1 h at room temperature. After each incubation step, the blots were washed three times with PBS-T buffer for 5 min each, followed by visualization using SuperSignal™ West Dura Extended Duration Substrate (ThermoFisher, Waltham, MA, USA). The CD63, CD9, and CD81 proteins were resolved under non-reducing conditions, where no DTT 0.1M is required to add to the samples.

### 2.10. Cryo-Electron Microscopy (Cryo-EM)

Cryo-electron microscopy (cryo-EM) was utilized to examine the morphology of small extracellular vesicles (sEVs) isolated via ultracentrifugation. In this procedure, approximately 4.5 μL of sEV sample (equivalent to around 10^6^ particles) was applied onto glow-discharged Quantifoil R2/2 copper grids (Quantifoil Micro Tools, Jena, Germany). The grids were then blotted for 2.5 s in a chamber maintained at 95% humidity before being plunged into liquid ethane using a Lecia EM GP device (Leica Microsystem, Wetzlar, Germany). Imaging was conducted using a Talos Arctica cryoTEM (Thermo Fisher Scientific, Waltham, MA, USA) operating at 200 kV, with the specimen maintained at liquid nitrogen temperatures. Images were captured at 28,000X magnification using a Falcon 3EC direct detector camera operated in linear mode.

### 2.11. RNA Extraction, cDNA Synthesis, and Digital PCR from sEV Samples

Isolation of total RNA from the sEVs was carried out using TRIzol LS according to the manufacturer’s instructions. The extracted RNA samples were then quantified using the NanoDrop™ One Microvolume UV-Vis Spectrophotometer (Thermo Scientific, Waltham, MA, USA), followed by cDNA synthesis using the High-Capacity cDNA Reverse Transcription Kit (4368813, Applied Biosystems, Foster City, CA, USA). Subsequently, digital PCR was performed utilizing the QIAcuity Probe PCR Kit (250102, Qiagen, Hilden, Germany) following the manufacturer’s protocol, with the same PCR conditions as mentioned above.

### 2.12. Statistical Analysis

Statistical analysis was performed using GraphPad Prism (ver. 8) to determine statistically significant upregulated circRNAs in the PM, normal mesothelial, and non-PM cell lines as well as the sEVs using a one-way analysis of variance (ANOVA). Results yielding a *p* value of <0.05 were considered statistically significant.

## 3. Result and Discussion

The sEVs exist in various body fluids, which is convenient for non-invasive detection [41]. CircRNAs are stable, conservative, and specific expression of cells and tissues, which suggests that they have the potential to be used as molecular diagnostic and prognostic markers [42]. sEV-derived circRNAs combine the advantages of using sEVs with the specificity of circRNAs, enhancing their potential application as early non-invasive biomarkers. sEVs derived from pathological cells can carry their disease-specific circRNA into the peripheral blood. Therefore, the detection of sEV-derived circRNAs in serum may be feasible in the diagnosis of tumor disease. CircRNAs have also been considered as EV biomarkers to monitor the progression and chemoresistance of some types of cancers. In addition, it has been discovered that circRNAs are stably expressed in sEVs and these circRNAs are suggested to be a promising candidate for biomarkers in cancer [35].

### 3.1. CircRNA Expression Profile in Mesothelioma

According to the results of our previous study [38], 290 circRNAs derived from host genes PHKB, SLC45A4, ARHGEF28, FBXW4, TAF15, PLEKHM1, RALGPS1, STIL, L3MBTL4, ANKRD27, NHS, ILKAP, and PTK2 in PM cell lines were upregulated using high throughput human lncRNA microarrays through fold change [38]. For the current study, we selected hsa_circ_0007386 (the one most highly expressed in four mesothelioma cells) as a representative circRNA for the PHKB gene [38] and investigated for changes in its expression levels to determine whether it could be employed as a potential biomarker for PM diagnosis.

### 3.2. sEV Characterization

**Figure 2:**
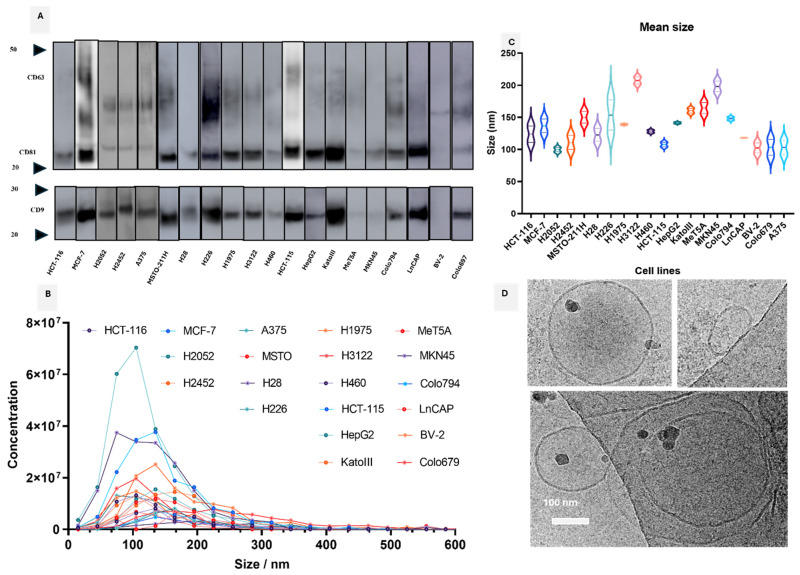
sEV characterization. (**A**) For Western blot analysis, sEVs were loaded on SDS-PAGE and immunoblotted for antibodies against tetraspanins (anti-CD9, anti-CD63, and anti-CD81). A gel was run under non-reducing condition with 2 × 10^8^ particles: ~5 µg. The exposure time was 35 s. (**B**) The concentration of isolated sEVs based on NTA analysis (all samples were diluted 1:100). (**C**) The size distribution of isolated sEVs showed sharp peaks between 100–200 nm. (**D**) Cryo-EM images of isolated sEVs from cell culture supernatant are shown (Scale bar: 100 nm). The extracted sEVs displayed perfect integrity with an average size of 100 nm.

For sEV characterization, particle size and concentration were evaluated by NTA, the morphology was evaluated with Cryo-EM and the expression of the sEV common protein markers (CD63, CD81, and CD9) was assessed using Western blotting (Figure 2). The tetraspanins (CD63, CD81, and CD9) were detected in the sEVs derived from the studied cell line using Western blotting (Figure 2A). The size distribution of sEVs, measured by NTA, and the concentration of sEVs enriched from cells is shown in Figure 2B. As shown in Figure 2C, most of the particles had a mean size of 100–200 nm. For further and more precise characterization of the sEVs, cryo-EM imaging was performed. Under cryo-EM, the specimens were imaged at extremely low temperatures (below −175 °C) so that sEVs retained their original spherical shape. The results of cryo-EM for sEVs confirmed their expected size and morphology (Figure 2D).

### 3.3. The hsa_circ_0007386 Highly Expressed in PM Cell Lines and sEVs

**Figure 3:**
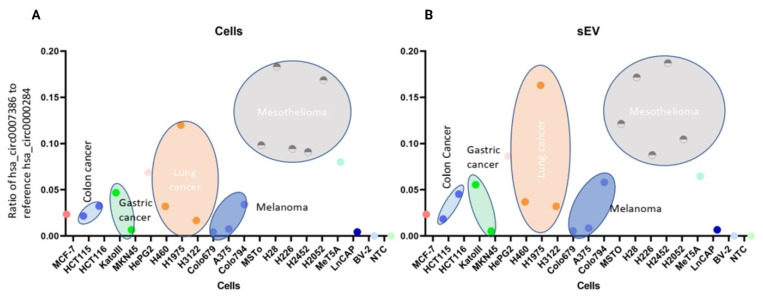
The copy number of specific hsa_circ_0007386 in (**A**) PM cell lines, and non-PM cell lines, compared to (**B**) PM cell derived sEVs, and non-PM cell derived sEVs using the digital PCR. Copy numbers of the circRNA were normalized using the internal reference control hsa_circ_0000284.

For the digital PCR analysis, the negative control for the hsa_circ_0007386 was used to adjust the threshold for achieving the correct signal from the positive samples, and the signal detected from the top right corner of the quadruplet was regarded as a positive control. In all samples, the background signal was used as the threshold above which signals were considered positive. Results of digital PCR revealed that among the studied cell lines, the hsa_circ_0007386 was overexpressed in NCI H-28 cells, with 1218.3 copies per µL input sample, followed by the H2052, H226, H2452, and MSTO-211H, with 696.7, 555.6, 412.5, and 187 copies per µL, respectively. The normal mesothelial cell line (MeT5A) showed 448 copies per µL. By normalizing the copy numbers using the ratio of hsa_circ_0007386 to hsa_circ_0000284, ratios were 0.183, 0.168, 0.098, 0.0943, and 0.0908 respectively (Figure 3A). Based on findings by Zhong et al. [43], hsa_circ_0000284 was selected as the internal control due to its demonstrated superior stability. This characteristic renders it an optimal candidate not only for circRNAs but also for broader RNA applications, serving as a reliable reference gene [43]. The results also indicate that the copy number ratio of hsa_circ_0007386 to the circ RNA reference was significantly lower in non-PM cell lines including melanoma (Colo794; 0.0340, Colo679;0.0042, A375; 0.00755), gastric (KATO III; 0.0467, MKN45;0.0066), lung (H460; 0.031, H3122; 0.0167), colon (HCT 115;0.0216, HCT 116;0.0323), breast (MCF-7; 0.0235), liver (HepG2;0.068), and prostate (LnCAP; 0.00433) cancer cell lines (*p* < 0.0001).

The hsa_circ_0007386 was overexpressed in H226 derived sEVs, with 36.23 copies per µL, followed by MSTO-211H, H2452, H2052, and NCI-H28, with 23, 11.71, 9.54, and 6.77 copies per µL of mixture, respectively. By normalizing the copy numbers found in sEVs using the hsa_circ_0007386 to hsa_circ_0000284 ratio, results differed from those attained using cells RNA extracts, and so did the ranking across cell lines as follows: H2452, NCI-H28, MSTO-211H, H2052, and H226 with the following ratios 0.187, 0.171, 0.121, 0.1045, and 0.087, respectively (Figure 3B). Interestingly, the copy number ratio of this circRNA biomarker to the circ RNA reference was significantly lower in non-PM cell lines including melanoma (Colo 794; 0.0580, Colo679; 0.0053, A375; 0.0084), gastric (KATO III; 0.055, MKN45; 0.0054), lung (H460; 0.036, H3122; 0.032), colon (HCT 116; 0.0452, HCT 115; 0.018), breast (MCF-7; 0.0233), and prostate (LnCAP; 0.0066) cancer cell lines (*p* < 0.0001). Indicating that the sorting of specific circRNA species to sEVs compared to the cells may be actively regulated.

**Figure 4:**
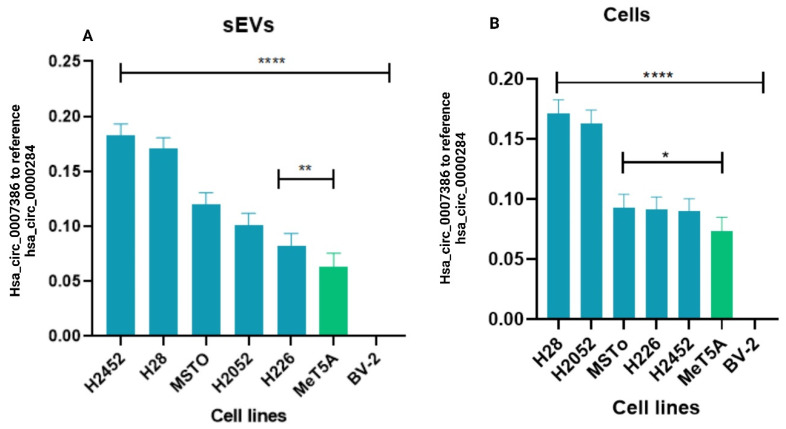
The copy number ratio of specific hsa_circ_0007386 to the reference circular RNA hsa_circ_0000284 in the (**A**) PM cell-derived sEV, and (**B**) PM cell lines compared to the normal mesothelial cell-derived sEVs (MeT5A) and normal microglial cell (BV-2)-derived sEVs and cell lines using the digital PCR. The copy number of circRNA was normalized using the internal reference control hsa_circ_0000284. In data analyses, the significance is shown by stars, the *p* value < 0.0001 is shown by ****, the *p* value 0.0017 is shown by **, and the *p* value 0.015 is shown by *.

The results of our study revealed the specificity of the hsa_circ_0007386 derived from both cells and sEVs as a specific biomarker for early diagnosis of PM, as we have observed significant expression of this biomarker in the PM cell-derived sEVs (Figure 4A) and PM cell lines (Figure 4B) compared to the normal mesothelial cell line (Met5A) and the microglial cell line (BV-2) (*p* < 0.0001).

The results of our study on PM and non-PM cells-derived RNAs confirm the 90% sensitivity and 93.3% specificity of the hsa_circ_0007386 as a biomarker in PM diagnosis. However, this number was 81.81% and 87.5% in the sEVs. The specificity was calculated by dividing the number of true negative samples, which are non-PM cell lines here, by the sum of true negative and false positive, which are the sum of non-PM cell lines here, and the cell lines that show a higher ratio of hsa_circ_0007386 to reference circRNA compared to the PM cell lines [44]. Similarly for the sensitivity calculation, the number of true positive samples, which are PM cell lines here, to the sum of true positive and false negative, which are the sum of PM cell lines here, and the cell lines that show a higher ratio of hsa_circ_0007386 to reference circRNA compared to the PM cell lines [44]. While our results demonstrate consistencies in the copy numbers or copy number ratios of hsa_circ_0007386 and hsa_circ_0000284 between PM and non-PM derived sEV samples and their corresponding cellular counterparts, the findings also reveal a higher copy number ratio of hsa_circ_0007386 to the reference hsa_circ_0000284 in sEV-derived samples compared to the cells. The heightened presence of hsa_circ_0007386 within PM-derived sEVs, compared to its cellular counterpart, underscores the propensity of circRNAs for encapsulation within sEVs. By scrutinizing the contents of sEVs, we can attain more precise and sensitive findings than those derived from the analyses of cellular extracts alone. Notably, our investigation revealed the maximal copy number of sEV-derived hsa_circ_0007386 within MSTO-211H, representative of the biphasic variant of PM. Early identification of this PM subtype holds substantial promise for advancing our understanding of disease progression. Consequently, the potential clinical value of sEV-derived hsa_circ_0007386 in less-invasive liquid biopsy approaches deserves consideration, with the outlook of replacing traditional tissue biopsies in the future. Our findings underscore the diagnostic specificity of hsa_circ_0007386 as a viable biomarker for early PM detection, with significantly elevated expression observed in PM cell lines compared to normal mesothelial, and other cancer cell lines (*p*-value < 0.0001). This highlights the promising diagnostic potential of hsa_circ_0007386 across both sEVs and cellular contexts. To ensure precision and reliability, further validation utilizing patient samples is imperative for consolidating these outcomes. In different stages of different development diseases, disease-related circRNA can be sorted into sEVs to be enriched and transported to target cells or target organs for release. Many studies have shown that differential expression of sEV-derived circRNAs in the body fluid was associated with the pathological characteristics of tumor vascular invasion, lymph node metastasis, poor survival, and Tumour, Node, Metastasis (TNM) stage [45,46,47]. Studies have shown that circRNAs can be packaged and function in sEVs [48]. However, the mechanism behind the selective packaging of specific circRNAs into sEVs is not yet clear and requires further investigation. In a study by Zhang et al. [49], it was shown that circRNA polo-like kinase 1 (circPLK1) was upregulated in Malignant Pleural Mesothelioma (MPM) tumor tissues and cell lines. CircPLK1 knockdown suppressed the proliferation, migration, invasion, and stemness of MPM cells in MPM progression. The studies mentioned have indicated the diagnostic efficacy of different circ-RNAs as a cancer marker.

**Figure 5:**
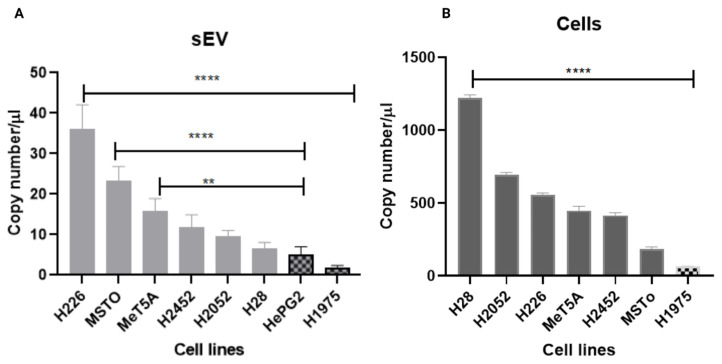
The copy number of specific hsa_circ_0007386 in the (**A**) PM cell-derived sEV, compared to the non-PM cell-derived sEVs (HepG2, and H1975) and (**B**) PM cell lines compared to the non-PM cell lines (H1975) using the digital PCR. The copy number of hsa_circ_0007386 in both PM cells and sEV-derived cells was significantly higher compared to the non-PM cell lines which showed a higher ratio of hsa_circ_0007386 to the reference hsa_circ_0000284. In data analyses, the significance is shown by stars, *p* value < 0.0001 is shown by ****, and *p* value < 0.0090 is shown by **.

In the PM cell-derived sEVs, a notable observation arose when comparing the copy number ratio of hsa_circ_0007386 to the circRNA reference across various PM cell-derived sEVs, particularly in the context of one lung cancer cell line (H1975) and one liver cancer (HepG2). Despite an apparently higher ratio in H1975 (0.1630) and HepG2 (0.086) compared to other PM cell lines (H20525, MSTO-211H, and H226), a deeper analysis utilizing raw data and absolute copy number calculations revealed that the observed copy number of this putative circRNA biomarker in the above-mentioned cell lines did exhibit statistical significance (*p* < 0.0001) (Figure 5A). The decision to employ exact copy number calculations was driven by the criterion of fewer than 100 copies per 40 µL of digital PCR sample, aiming to enhance result accuracy [50]. Similarly, in the PM cell lines, comparable to our observations in the cell-derived sEVs, in one lung cancer cell line (H1975) despite an apparently higher ratio of hsa_circ_0007386 to the circRNA reference (0.12) compared to other PM cell lines (MSTO-211H, H226, and H2452), a deeper analysis utilizing raw data and absolute copy number calculations revealed that the observed copy number of this putative circRNA biomarker in the H1975 cell line did exhibit statistical significance (*p* < 0.0001) (Figure 5B).

Results of a systematic review and meta-analysis revealed that circRNAs have the potential to be biomarkers for diagnosis and prognosis of cancers [51]. It has been described by Stella et al. that two circRNAs localized in serum-derived sEVs including circSMARCA5 (hsa_circ_0001445) and circHIPK3 (hsa_circ_0000284) could be potential biomarkers for glioblastoma and could distinguish glioblastoma patients from healthy controls with high accuracy [52]. In contrast, another study has reported that the continued high expression of sEV-derived circRNA-100338 in the serum of HCC (Hepatocellular Carcinoma) patients undergoing therapeutic hepatectomy may be related to lung metastasis and poor survival [53].

SEV-derived circRNAs are critical regulators of both healthy and diseased states, and they may represent valuable biomarkers for the diagnosis and prognosis of multiple cancers. The potential of sEV-derived circRNAs as biomarkers for lung cancer [54], gastric cancer [55], colorectal cancer [56], and hepatocellular carcinoma [57] have been reported. Currently, multiple diagnostic clinical trials are in progress, aiming to validate the utility of emerging sEV-based biomarkers for diagnosis, prognosis, and treatment prediction across various cancer types. However, there is a critical need for more randomized clinical trials in this field. Such studies are essential for identifying patients with specific immune responses to cell injury, thereby advancing the precision medicine approach in oncology. By focusing on these trials, the potential of sEV-based biomarkers could be fully realized, paving the way for more personalized and effective cancer treatments [58].

There are some advantages in analyzing sEV-encapsulated non-coding RNAs compared to whole plasma/serum. Firstly, as extracellular vesicles can be secreted by a variety of cells, the contents of sEVs can be used as biomarkers for diagnosis or prognosis in various diseases [59]. Secondly, it is easier to sort circRNA into sEVs than linear RNAs [35]. In addition, sEVs derived from cancers contain highly specific RNA, and they can also prevent nucleic acid molecules from degradation by RNase in the blood [60]. However, there are still many issues to be resolved before sEVs-derived circRNAs can be employed as reliable biomarkers, such as preservation of specimens, cell source of sEVs, sEVs isolation methods, etc.

Future investigations focusing on sEV circRNAs in various biological contexts, such as the hematopoietic system, immune response, nervous disorders, cancer development, and other diseases, will provide further insights into the enigmatic nature of sEV circRNAs. Consequently, elucidating the mechanisms of cancer pathogenesis and identifying potential novel diagnostic biomarkers or therapeutic targets are expected to be prominent areas of research in the future.

## 4. Conclusions

In this study, the sEV-derived hsa_circ_0007386 was found to be an effective and novel biomarker for pleural mesothelioma, suggesting that they may be involved in the occurrence and development of PM. This mechanism remains to be further explored. This study has the potential to be used in clinical applications based on less-invasive liquid biopsy, which will be able to replace conventional tissue biopsies in the near future and provide novel potential treatment options for mesothelioma patients. The finding has significant implications for the diagnosis and treatment of PM, which currently has a poor prognosis after diagnosis. This outcome would be particularly beneficial for the quality of life of PM patients, as there would be no associated post-surgery recovery burden. Furthermore, the rapid processing time associated with sEV-based biomarkers would ensure that PM patients receive prompt treatment.

## Figures and Tables

**Table 1:** List of primer and probes used for the detection of hsa_circ_0007386 in human-derived cells and sEVs RNA.

Name	Sequence 5′-3′
hsa_circ_0007386 (Forward)	CGG ACA GCT ATG AAA CTC AA
hsa_circ_0007386 (Reverse)	CTT CAC AGC GGG CCT T
hsa_circ_0007386 FAM (Probe)	ACT TTG CCA ACA AGA AGA G
hsa_circ_0000284 (Forward)	CGG CCA GTC ATG TAT CAA A
hsa_circ_0000284 (Reverse)	TTC TTC ACA CTA CAA AAG GCA
hsa_circ_0000284 HEX(Probe)	TCG GTA CTA CAG GTA TGG CTC ACA

## Data Availability

All datasets are presented in the main manuscript as well as in the supporting file and there would be no additional data to be deposited in any dataset.
